# Choices and Challenges With Drug Therapy in Postural Orthostatic Tachycardia Syndrome: A Systematic Review

**DOI:** 10.7759/cureus.38887

**Published:** 2023-05-11

**Authors:** Advait M Vasavada, Deepak Verma, Vineetha Sheggari, Srushti Ghetiya, Punith Chowdary Chirumamilla, Radhika A Kotak, Shruti Sagar Mahapatra, Tirath Patel, Manisha Jain

**Affiliations:** 1 Internal Medicine, MP Shah Medical College, Jamnagar, IND; 2 Internal Medicine, Sri Guru Ram Das Institute Of Medical Sciences and Research, Amritsar, IND; 3 Internal Medicine, Dr Vizarath Rasool Khan (VRK) Women’s Medical College, Hyderabad, IND; 4 Internal Medicine, Gujarat Medical and Education Research Society (GMERS) Medical College, Junagadh, IND; 5 Internal Medicine, Guntur Medical College, Guntur, IND; 6 Internal Medicine, DY Patil University, School of Medicine, Navi Mumbai, IND; 7 Internal Medicine, Srirama Chandra Bhanja (SCB) Medical College and Hospital, Cuttack, IND; 8 Surgery, American University of Antigua, St John's, ATG; 9 Internal Medicine, Shri Bhausaheb Hire Government Medical College, Dhule, IND

**Keywords:** pots-all types, treatment, drug therapy, orthostatic intolerance, postural orthostatic tachycardia syndrome

## Abstract

The literature on pharmacologic treatments for postural orthostatic tachycardia syndrome (POTS) is inconsistent and unstandardized. Therefore, we aimed to evaluate choices in pharmacologic treatment options for POTS and the challenges encountered in the studies. We searched numerous databases like PubMed, Scopus, Embase, Web of Science, and Google Scholar for literature published before April 8, 2023. The search was done to retrieve potential peer-reviewed articles that explored drug therapy in POTS. Preferred Reporting Items for Systematic Review and Meta-Analysis (PRISMA) guidelines were used to conduct the systematic review. Of the 421 potential articles assessed, 17 met the inclusion criteria. Results demonstrated that pharmacologic treatment options for POTS were effective in reducing symptoms of POTS, but most of the studies were underpowered. Several were terminated due to various reasons. Midodrine ivabradine, bisoprolol, fludrocortisone, droxidopa, desmopressin, propranolol, modafinil, methylphenidate, and melatonin have been studied with positive impact but sample sizes that were low in the range of 10-50 subjects. Therefore, we concluded the treatment options effectively improve symptoms of POTS and increase orthostatic tolerance, but more evidence is needed as most studies had a low sample size and thus are underpowered.

## Introduction and background

Postural orthostatic tachycardia syndrome (POTS) is a chronic orthostatic intolerance that is considered to be an autonomic dysfunction characterized by the inability of the involuntary nervous system to properly control blood flow, gastrointestinal flow, and body temperature [1,2]. This results in postural symptoms like persistent fatigue, dizziness, lightheadedness, and palpitations, especially upon standing [3]. The prevalence of POTS is proximately between 0.2% and 1% of the population, most commonly diagnosed in women between the ages of 15 and 50. Some studies suggest that the prevalence of POTS may be higher in certain populations, such as people with chronic fatigue syndrome or autoimmune disorders. POTS can occur on its own, or it can be associated with other medical conditions such as Ehlers-Danlos syndrome, Lyme disease, COVID-19, or autoimmune disorders [3,4].

**Figure 1:**
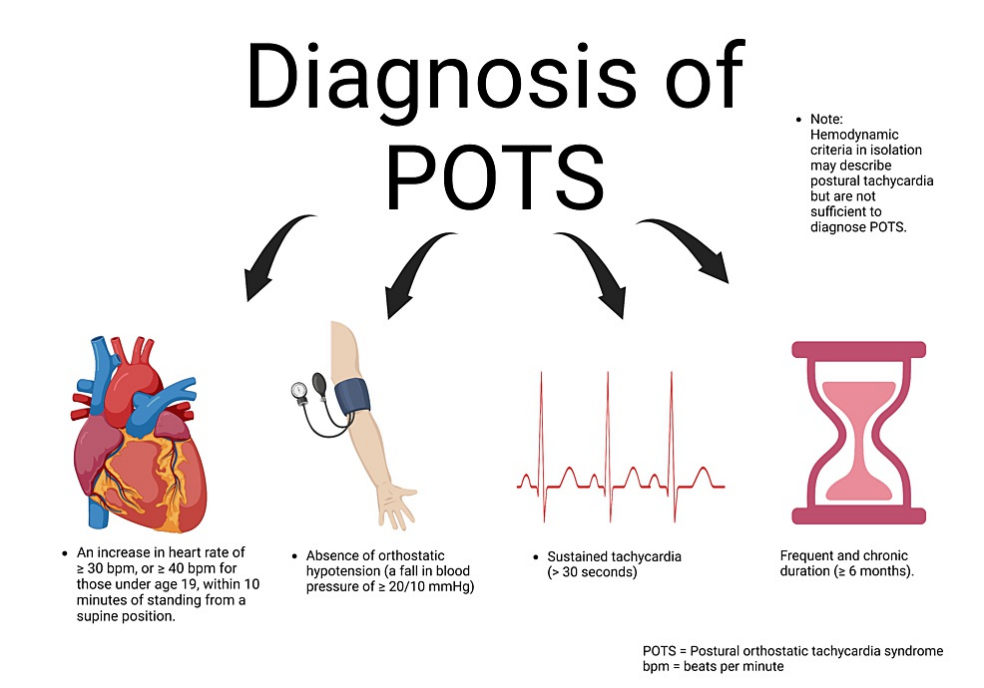
Diagnosis of POTS POTS: postural orthostatic tachycardia syndrome, bpm: beats per minute Source: Advait Vasavada (created with Biorender.com (Science Suite Inc, Canada), agreement number UE25C1X64H)

The diagnosis is based on the presence of symptoms of orthostatic intolerance that occur upon standing and are relieved while lying down [5]. This is coupled with an abnormal increase in standing heart rate as evaluated by a head-up tilt test. To meet the criteria for diagnosis, the symptoms should have existed for a chronic duration and a minimum heart rate change of 30 beats per minute in patients aged 18 years or more and 40 beats per minute in adolescents within 10 minutes of upright posture [6]. Figure 1 highlights the definition/diagnostic criteria.

The disease is a complicated multi-system disorder contributing to poor quality of life [7]. Pharmacologic therapies have been explored in minimizing symptoms and increasing functional ability in POTS patients [2]. Treatment targets have focused on reducing heart rate (beta-blockers), increasing cardiac output (increase in salt intake), or increasing blood pressure (midodrine). The therapies are based on the hypothesis that affecting the said parameters will reduce symptom burden and are aimed at managing symptoms such as lightheadedness, dizziness, and rapid heartbeat that occur when standing up [2]. POTS patients face considerable physical, psychosocial, and financial challenges in the absence of a definitive cure [8].

It is essential to tailor treatment approaches to each person's unique clinical presentation, comorbidities, and drug tolerance. Complementary therapies that do not include medication use include cognitive behavioral therapy and compression stockings [9]. When prescribing medicine for POTS patients, clinicians should also be mindful of the risks of side effects and combinations with other drugs. Prior studies have revealed the therapies are inconsistent and unstandardized. The objective of this systematic review was to evaluate the challenges in the development of drug therapy and evaluate options in the management of POTS by synthesizing the available evidence.

## Review

Methods

Preferred Reporting Items for Systematic Review and Meta-Analysis (PRISMA) guidelines [10] were utilized to find published studies on the pharmacologic treatment options for postural tachycardia syndrome. Studies fulfilling the revised Population, Intervention, Comparison, Outcomes, and Study (PICOS) design criteria were selected [11]. The PICOS criteria for eligible studies were defined as follows: Population (P): All patients are involved in assessing the efficacy of common pharmacologic treatment options for postural tachycardia syndrome. Intervention (I): Medications such as beta-blockers, fludrocortisone, midodrine, and ivabradine. Comparison (C): Cardiovascular parameters and symptom burden in intervention group Vs. In the control group. Primary outcomes (O): Cardiovascular parameters such as standing heart rate and blood pressure. Secondary outcomes: Symptom burdens such as dizziness, lightheadedness, and fatigue. Study design (S): Randomized controlled trials, case series, and retrospective (observational) studies were included.

Search strategy

Eligible studies included in this review had to meet the following inclusion criteria: journal articles; published in the English language; studies that explored the treatment options available and challenges with the evidence related to the treatment options for postural tachycardia syndrome; and studies with randomized controlled trials (RCTs), case series, and retrospective observational studies. Ineligible studies were excluded due to the following reasons: narrative/systematic reviews, case reports, meta-analyses, protocol papers, bibliometric papers, opinion pieces, abstracts, or editorial articles that have no methods and results and explored the efficacy of non-medication treatments for postural tachycardia syndrome.

A detailed systematic literature search of PubMed, Scopus, Embase, and Web of Science was conducted as principal sources and Google Scholar as a supplemental source. This search was done for articles published from January 1, 2000, to April 8, 2023. The search was done to retrieve potential peer-reviewed articles (RCTs, case series, and retrospective observational studies) that explored the effect of common pharmacologic treatment options in POTS. The following keywords and medical sub-headings were used in the search: ("Postural Orthostatic Tachycardia Syndrome" OR "Postural tachycardia syndrome" OR "Orthostatic Tachycardia Syndrome") AND (efficacy OR effectiveness) AND (Therapy OR Pharmacotherapy OR Treatment). Synonyms and alternative spellings were also used. The keywords were used exhaustively in different combinations in different electronic databases. The snowballing technique was used to explore any missed studies. Two independent reviewers (A.V. and D.V.) screened the titles and abstracts of the retrieved articles based on the predefined inclusion and exclusion criteria. Full-text articles of potentially relevant studies were retrieved for further assessment. Any discrepancies were resolved through discussion and consensus by a third party.

Data extraction

After completing the preliminary search strategy, a study was chosen through a systematic approach. The Zotero reference manager (Corporation for Digital Scholarship, Virginia, USA) was used to eliminate duplicate references, and the remaining studies were subjected to screening. All articles that had the potential to meet the inclusion criteria were reviewed. In order to remove unsuitable studies, the titles and abstracts of the articles were initially screened. The full texts of the remaining studies were then assessed to determine whether they met the current inclusion and exclusion criteria. A meticulously designed form with important search terms was used to extract data. The accuracy of relevant studies was evaluated. Eligible studies that met the inclusion were critically checked for quality. The Risk of Bias 2 (RoB 2) tool (Cochrane, Northern Ireland, UK) was used to independently assess and confirm the accuracy of included RCTs [12]. RoB 2 uses the following bias criteria: bias arising from the randomization process, bias due to deviations from intended intervention, bias due to missing outcome data, bias in the measurement of the outcome, bias in the selection of the reported result, and overall risk of bias. The judgment is either low, high, with some concerns, or no information bias. Non-controlled trials were assessed using the modified Newcastle-Ottawa Scale [13]. This scale uses selection, ascertainment, causality, and reporting bias. In our review, there were 12 articles that were RCTs and five that were non-RCTs (retrospective observational studies/case series).

Results

**Figure 2:**
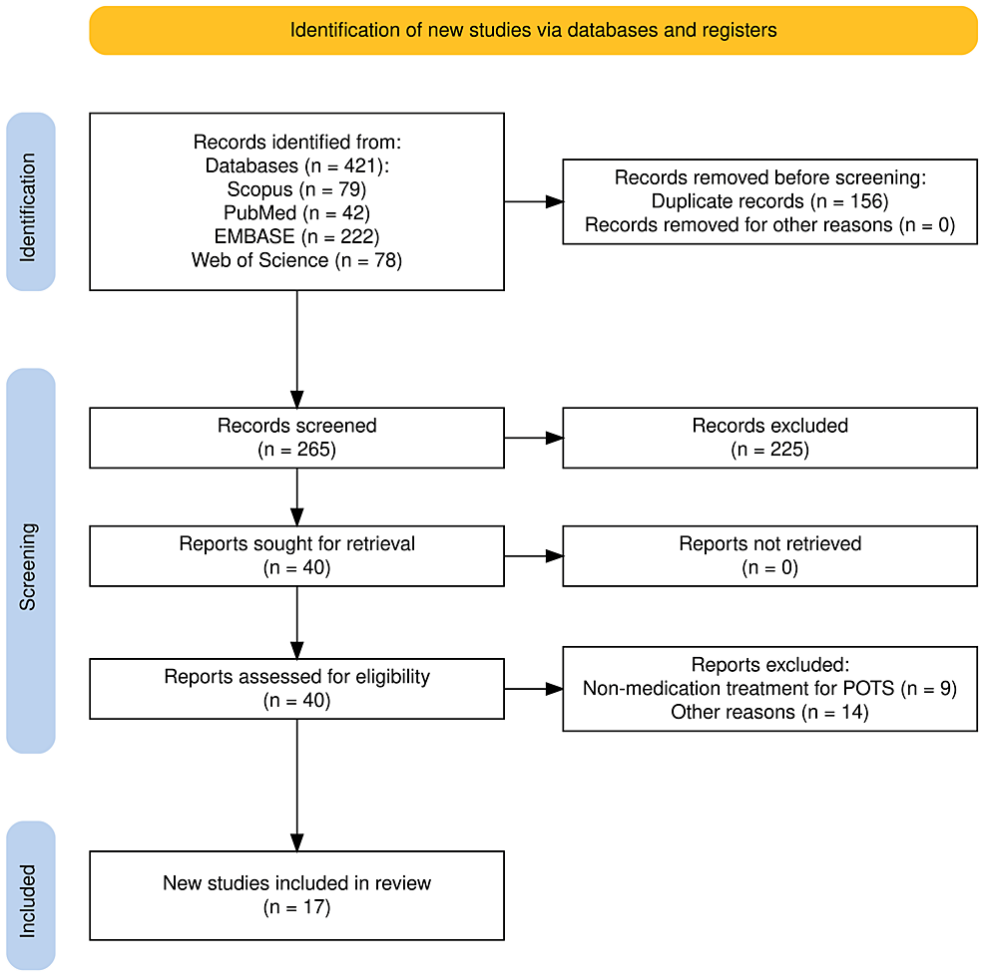
PRISMA chart Created using the Shiny app by Haddaway et al. (2022) [14].

Database search yielded 421 articles, out of which 156 duplicates were eliminated. The title and abstract screening removed 225 articles. Forty articles were sought for retrieval and subsequently assessed for eligibility. Twenty-three articles were eliminated as they did not meet the stipulated inclusion criteria. Seventeen articles were found to be eligible for review after the screening, as indicated in Figure 2 in a PRISMA chart created using the Shiny app by Haddaway et al. (2022) [14].

Data synthesis

In the studies, the symptom burden was measured and analyzed using patient-reported outcome measures (PROMs) and physiological measurements. PROMs used in the study included the Orthostatic Grading Scale, which assesses the severity of orthostatic symptoms, and the Composite Autonomic Symptom Scale (COMPASS), which measures autonomic symptoms such as lightheadedness, palpitations, and sweating. Patients completed these questionnaires before and after treatment. High scores indicated a greater symptom burden and poor quality of life. Additionally, heart rate was measured using ECG at baseline and regular intervals after treatment. Neuropathic POTS is caused by dysfunction or damage to the autonomic nervous system and is often associated with conditions such as diabetes, autoimmune disorders, or genetic disorders that affect the autonomic nervous system. In contrast, hyperadrenergic POTS is characterized by an overactive sympathetic nervous system and is linked with conditions such as Ehlers-Danlos Syndrome or a history of trauma, infection, or surgery. Neuropathic POTS may benefit from medications that improve autonomic function, while hyperadrenergic POTS may benefit from medications that decrease sympathetic activity, but currently, no guidelines exist. The control group in most of the studies were patients with POTS who were prescribed a placebo or an alternative drug.

The characteristics (author, publication year, sample size, age range, study design, medication outcome, and major findings) were extracted and summarized in Table 1.

**Table 1:** Study characteristics Studies [7,9,15-29]. CVR: calf vascular resistance, Qcalf: calf blood flow, Cv: calf venous compliance, QoL: quality of life, BP: blood pressure, HR: heart rate, H2S: hydrogen sulfide

Author (year)	Study design	Sample size	Mean age/range	Medication/control	Outcome	Major finds
Ross et al. (2014) [15]	Randomized controlled cross-over	20:15F,5M	12 to 20	Midodrine/placebo as control	Increase in CVR and decrease in Qcalf and Cv for patients with neuropathic phenotype	Midodrine: Showed promise in neuropathic but not hyperadrenergic type of POTS
Freitas et al. (2000) [9]	Observational study	11F	31 ± 11	Bisoprolol and/or fludrocortisone	Patients were symptomatically better after drug therapy	Outcomes with autonomic and hemodynamic impairment showed promise with bisoprolol or fludrocortisone or both
Coffin et al. (2012) [7]	Randomized cross-over	30:26F,4M	37 ± 12	Desmopressin (DDAVP)/placebo as control	Standing heart rate was lower following DDAVP vs. placebo (101.9 14.5 beats/min vs. 109.2 17.4 beats/min)	Oral DDAVP improved tachycardia and symptoms in POTS
Ruzieh et al. (2017) [16]	Retrospective study	37:28F,11M	48.08 ± 18.1	Droxidopa/no control	Symptoms of dizziness, syncope, and fatigue were reported reduced after drug therapy; 75.7%, 51.4%, and 40.5%, respectively. No difference in standing or sitting blood pressure before and after treatment	Droxidopa led to less orthostatic intolerance but had minimal impact on QoL and BP
Chen et al. (2008) [17]	RCT	55:32F,23M	12 ± 3.1	Midodrine hydrochloride+ oral rehydration and salt treatment/control as oral rehydration salt treatment only	Pharmacotherapy led to symptom improvement compared to control after three and six weeks of treatment. Higher disease-free rate at the follow-up end-point in the treatment group vs control	Selective alpha-1 receptor agonist midodrine hydrochloride effectively treats children with POTS
Moon et al. (2018) [18]	RCT	77:41F,36M	33.0 ± 12.7	Four treatment groups (Group 1: propranolol; Group 2: bisoprolol; Group 3: propranolol + pyridostigmine; Group 4: bisoprolol + pyridostigmine). 2x2 factorial design RCT	The OIQ score improved in every treatment arm. Subgroup analysis of 59 patients not receiving antidepressants concluded that the BDI-II scores reduced after drug therapy (all regimens). SF-36 was noted to have improved physical components after three months in all groups	Sustained medical treatment benefits patients. All outcomes like orthostatic intolerance symptoms, depression, and quality of life improved even without the need for antidepressants
Kpaeyeh et al. (2014) [19]	RCT	54:48F,6M	32 ± 10	Modafinil/placebo as control	No difference in standing HR between the modafinil and placebo groups	Modafinil could be a potential treatment for cognitive impairment in POTS
Green et al. (2014) [20]	RCT	78:72F,6M	32 ± 9	Melatonin/placebo as control	No difference between melatonin and placebo for decreasing BP (standing or while seated). No symptom burden improvement	Oral melatonin led to some decrease in standing tachycardia in POTS. Regular night-time use of this medication in POTS yet to be properly evaluated
Chen et al. (2011) [21]	RCT	53:22M,32F	12.2 ± 2.4	Group I (midodrine hydrochloride plus conventional therapy), Group II (metoprolol plus conventional therapy), and Group III (conventional therapy)	Group I had a notably lower rate of recurrent symptoms compared to Group II and Group III (P<0.05). However, there was no significant difference in the recurrence rate between Group II and Group III (P>0.05)	Midodrine hydrochloride had a favorable effect on children with POTS
Raj et al. (2009) [22]	RCT	72:65F,7M	33 to 34	Protocol 1: Propranolol/placebo as a control. Protocol 2: High dose vs low dose propranolol	Propranolol exhibited a more substantial improvement in symptom burden from baseline to two hours than placebo	In POTS, low-dose oral propranolol was effective in reducing tachycardia and improving symptoms, while higher doses did not offer further benefits and could potentially worsen symptoms
Taub et al. (2021)[23]	RCT	37:35F,2M	33.9 ± 11.7	Ivabradine/placebo as control	Heart rate showed a significant decrease between placebo and ivabradine groups (p<0.001). Patients also reported notable enhancements in their quality of life based on the RAND 36-Item Health Survey 1.0, particularly in physical functioning (p = 0.008) and social functioning (p = 0.021)	For patients with hyperadrenergic POTS as the predominant subtype, ivabradine is a secure and efficient treatment option for significantly improving heart rate and QoL
Yozgat et al. (2020) [24]	RCT	70	13.26 ± 2.55	Propranolol and oral rehydration in the study group, no medication in the control group	Following the treatment, there was a significant reduction in the frequency of syncopal attacks for both groups (P<0.01 for both). However, in the pediatric study group, the post-treatment standardized symptom scores were significantly lower than those of the control group	The control group continued to experience symptoms. Based on its clinical effectiveness, we strongly recommend the combined treatment of reduced-osmolarity ORS and low-dose propranolol for pediatric patients diagnosed with POTS
McDonald et al. (2011) [25]	Retrospective study	22	35 ± 9.9	Ivabradine	Eight individuals reported a reduction in both tachycardia and fatigue, while four reported only a reduction in tachycardia. The primary reason for discontinuing ivabradine treatment was due to its lack of efficacy (n = 6). Five patients reported experiencing side effects, leading to two individuals discontinuing the treatment	According to this retrospective case series, 60% of patients who received ivabradine treatment reported experiencing an improvement in their symptoms
Hoeldtke et al. (2006) [26]	RCT	15	36.3	Combination therapy vs monotherapy	The standing time for patients with OI, initially recorded as 36.3 ± 3.5 minutes, showed an improvement with the use of midodrine, octreotide, and combination therapy. Before the treatment, the standing heart rate for OI patients was measured at 100 ± .76 bpm, but after administering midodrine, it decreased to 80.3 ± .69 (P<0.05). Similarly, following octreotide, the standing heart rate was 84.8 ± .86, and after combination therapy, it decreased to 71.2 ± .9 (P<0.01)	In POTS, midodrine and octreotide effectively controlled tachycardia and improved standing times in OI patients. The two drugs exhibited comparable effectiveness, and there was no significant advantage of combination therapy over monotherapy
Kanjwal et al. (2012) [27]	Retrospective	24:20F,4M	28 ± 12	Methylphenidate	Among the eighteen patients, fourteen individuals (77%) reported a significant improvement in their symptoms, including fatigue and presyncope. Furthermore, out of the 12 patients with recurrent syncope episodes, nine individuals reported no syncope after six months of follow-up	Methylphenidate may be of use in patients with refractory postural tachycardia syndrome
Towheed et al. (2020) [28]	Retrospective	27:25F,2M	12 ± 17	Ivabradine	Out of 27 patients, symptoms improved in 18 individuals (67%). The symptom that showed the most significant improvement was syncope/presyncope, which was reduced by 90%, followed by lightheadedness (85%) and fatigue (81%)	The findings of this study suggest that 67% of children who received ivabradine treatment reported experiencing an improvement in their symptoms
Yang et al. (2013) [29]	RCT	28:12M,16F	11.5 ± 2.5	Midodrine hydrochloride in patients with POTS/healthy children without medication as the control group	The POTS group demonstrated a noteworthy increase in H2S production from erythrocytes compared to the control group, with a statistical significance of P<.01 moreover individuals who responded to midodrine hydrochloride showed significantly higher h2s production compared non-responders with a significance of p	H2S produced by erythrocytes may serve as a valuable predictor for the therapeutic response to midodrine hydrochloride in pediatric patients diagnosed with POTS

**Figure 3:**
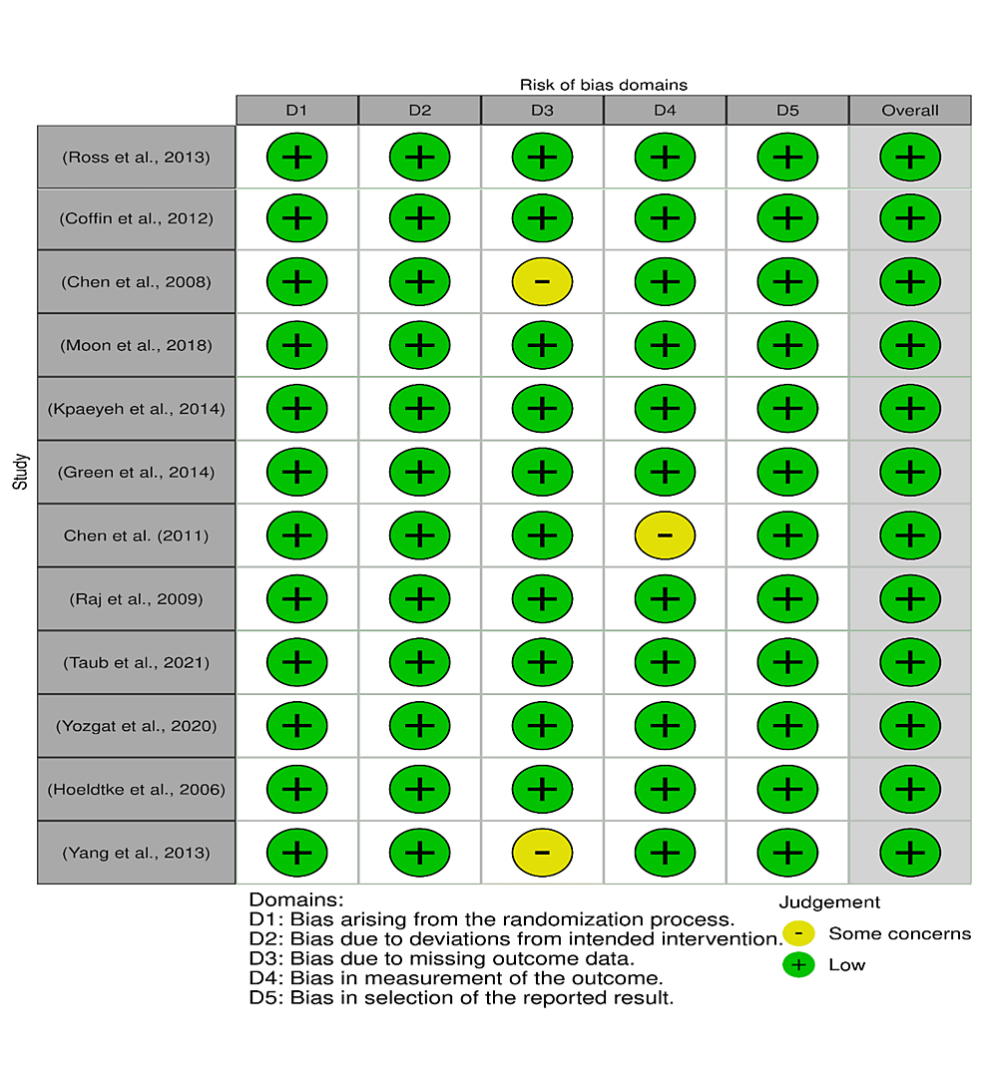
Traffic light plot highlighting the quality appraisal for the studies Studies [7,15,17-24,26,29]

See Figure 3 for the quality appraisal done for all the studies.

Table 2 shows the NOS scale used for quality assessment for the rest of the studies.

**Table 2:** NOS for quality appraisal Studies [9,16,25,27,28]

Author (year)	Selection	Ascertainment		Causality				Reporting
	Q1	Q2	Q3	Q4	Q5	Q6	Q7	Q8
Ruzieh et al. (2017) [16]	No	Yes	Yes	No	No	No	Yes	Yes
McDonald et al. (2011) [25]	No	Yes	Yes	No	No	No	Yes	Yes
Kanjwal et al. (2012) [27]	No	Yes	Yes	No	No	No	Yes	Yes
Towheed et al. (2020) [28]	No	Yes	Yes	No	No	No	Yes	Yes
Freitas et al. (2000) [9]	No	Yes	Yes	No	No	No	Yes	Yes

Discussion

A constructive compilation of studies conducted on drug treatment options for POTS reveals studies with a range of sample sizes between 20 and 80. Although there was no bias in the trial methodology or protocol, these studies have a smaller sample size and, thus, are underpowered. The pharmacologic treatments options for postural tachycardia syndrome include midodrine ivabradine, bisoprolol, fludrocortisone, droxidopa, desmopressin, propranolol, modafinil, methylphenidate, and melatonin. Patients with POTS may respond differently to therapy depending on their intensity, underlying disorders, and other medical conditions [2]. The treatment is directed at symptom burden which may or may not include dizziness/lightheadedness, fainting, rapid heartbeat/palpitations, chest pain/discomfort, shortness of breath, headaches, fatigue/weakness, nausea, abdominal pain, brain fog/difficulty concentrating, blurred vision, sweating, shakiness/tremors, insomnia/disrupted sleep, exercise intolerance. Earlier systematic reviews and meta-analyses have explored this aspect where pre-pandemic there were only three randomized controlled trials included in their analysis [30]. Even today, the trials conducted are sparse and several have been reported to be terminated [31,32].

Given the underpowered studies, we evaluated the studies based on the Grading of Recommendations, Assessment, Development, and Evaluations (GRADE) framework and illustrated the certainty in evidence [33]. The certainty ratings have four levels: very low, low, high, and very high. Figure 3 demonstrates GRADE ratings for studies included in our review.

**Table 3:** GRADE certainty ratings Studies [7,9,15-29]

Study	GRADE certainty rating
Ross et al. (2014) [15]	Very low
Freitas et al. (2000) [9]	Very low
Coffin et al. (2012) [7]	Low
Ruzieh et al. (2017) [16]	Low
Chen et al. (2008) [17]	Low
Moon et al. (2018) [18]	Low
Kpaeyeh et al. (2014) [19]	Low
Green et al. (2014) [20]	Low
Chen et al. (2011) [21]	Low
Raj et al. (2009) [22]	Low
Taub et al. (2021) [23]	Low
Yozgat et al. (2020) [24]	Low
McDonald et al. (2011) [25]	Very low
Hoeldtke et al. (2006) [26]	Very low
Kanjwal et al. (2012) [27]	Very low
Towheed et al. (2020) [28]	Very low
Yang et al. (2013) [29]	Very low

Challenges with studying POTS

Our review concluded that studying POTS in RCTs has been very challenging. POTS prevalence has been difficult to determine because of its heterogeneous nature, and it is challenging to recruit a sufficient number of participants for clinical trials as exemplified by the study characteristics table and the terminated trial data [31,32,34]. Small sample sizes can limit the statistical power of the study and increase the risk of a type II error, which occurs when a study fails to detect a true effect. POTS can have various underlying causes, and the symptoms and severity of the condition can vary widely between patients. This heterogeneity can make it challenging to identify a homogeneous patient population for a clinical trial and can also complicate the interpretation of study results [30]. In addition, POTS symptoms are highly variable and subject to placebo effects leading to confusion. Some studies have reported high placebo response rates, which can obscure the effects of the investigational treatment. There is also no consensus currently on the most appropriate outcome measures for POTS clinical trials. All this is making it challenging to compare results across studies and hinders the development of new treatments. Some POTS clinical trials have been terminated early due to safety concerns or adverse events associated with the drug therapy (unexpected side effects or adverse reactions that are not well understood) [31,32,34].

Despite these challenges, there is a growing interest in POTS research, and ongoing efforts are being made to improve diagnostic criteria and develop new treatments. Clinical trials are an essential part of this effort and will be necessary to advance our understanding of the condition and develop effective treatments. On top of that, we suggest several measures that could help address the problem of early termination of clinical trials.

Measures to avoid trial termination

The use of patient advocacy groups can help to identify potential participants and raise awareness of the study. They can also provide valuable feedback on study design and outcome measures, which can help to ensure that the study is relevant to patients' needs [35]. Another strategy is to conduct feasibility assessments before launching a trial, which can help identify potential issues and reduce the risk of early termination. Feasibility assessments can help to determine whether the patient population is available and willing to participate, whether the trial sites have adequate resources, and whether the study design and outcome measures are feasible. The use of adaptive trial designs can help to reduce costs and improve the efficiency of clinical trials [36]. Adaptive designs allow for modifications to the trial protocol based on interim data analysis, which can help to reduce the sample size and shorten the duration of the trial. A focus on patient-reported outcomes can be used to assess the impact of POTS on patients' quality of life and to evaluate the effectiveness of treatments. Using patient-reported outcomes can help to reduce the burden of the trial on patients and make the study more relevant to their needs [37]. Traditional trial endpoints, such as symptom improvement or physiological measures, may be difficult to achieve in POTS due to the variability and heterogeneity of the condition. Consideration should be given to alternative endpoints such as functional improvement or quality-of-life measures that may better reflect the impact of the treatment on patients. By incorporating these strategies into trial design, it may be possible to improve the efficiency and cost-effectiveness of clinical trials while minimizing the risk of early termination [38].

Focus on long-term outcomes

POTS can be chronic and debilitating. Hence, prospective trials should also focus on long-term outcomes like quality of life, activity tolerance, syncopal episodes, sleep quality, and medication use. The more homogenous the outcome measures, the better we will be able to analyze the data. Quality of life can be assessed using standardized questionnaires such as the Short Form-36 or the EuroQol-5 Dimension [39]. These questionnaires assess various aspects of quality of life, including physical and emotional functioning, social relationships, and overall well-being. Mobility and activity tolerance can be evaluated using objective measures such as the six-minute walk test or the exercise capacity test. Syncope and near-syncope episodes can be tracked using patient diaries or event monitors that record the frequency and duration of these episodes. Heart rate and blood pressure monitoring using accurate monitoring devices, including ECGs and blood pressure monitors, can aid in this. Wearable device technology should definitely be explored. Sleep quality can be assessed using questionnaires such as the Pittsburgh Sleep Quality Index (PSQI) or objective measures such as actigraphy, which measures movement during sleep [40,41]. Medication use can be evaluated using patient diaries or by recording the dose and frequency of medications used to manage POTS symptoms. These methods to measure long-term outcomes can assess the effectiveness of interventions for improving POTS symptoms and provide evidence to support the development of effective treatments for this condition.

Evidence shows that various treatments for POTS symptoms may work better than others [2,30,42]. In our review, we have not included any non-pharmacological interventions that are usually recommended first, for example, increased salt intake [30]. This review found that midodrine was the most studied and effective medicine for reducing orthostatic symptoms, including dizziness and fainting. Additionally, midodrine helped patients with neuropathic and hyperadrenergic POTS by decreasing orthostatic hypotension and increasing cerebral blood flow [15]. Treatment with bisoprolol and fludrocortisone alleviated clinical symptoms in individuals with orthostatic intolerance [9]. Reducing heart rate and alleviating symptoms like palpitations and tremors were both achieved with the use of beta-blockers. Beta-blockers may not be the best choice for people with low blood pressure as it could potentially worsen their condition. Evidence for the use of ivabradine in POTS is scant. However, it was shown to be useful in lowering heart rate and relieving symptoms. Although pyridostigmine was proven to alleviate symptoms, insufficient data supports its usage in children and adolescents. In individuals with POTS, lowering the standing heart rate is crucial because it may help reduce symptoms, including lightheadedness, dizziness, and fainting caused by an excessively high heart rate upon standing [43]. Patients with POTS may significantly improve their symptoms and quality of life if they can slow their heart rate while standing. Long-term issues, including heart failure and other cardiovascular disorders, may also be caused by an unusually high heart rate. The potential consequences of POTS may be reduced with proper heart rate management [7].

The reduction in symptom burden may be due to the ability of pharmacologic treatments to regulate the autonomic nervous system, which controls many of the body's involuntary functions, including heart rate, blood pressure, and digestion. By regulating the autonomic nervous system, pharmacologic treatments can help improve blood flow and reduce symptoms such as lightheadedness, dizziness, and fatigue, common in patients with POTS [29,44]. Patients and doctors alike face formidable obstacles in dealing with postural tachycardia syndrome. Because POTS may profoundly influence patients' quality of life, there is an urgent need for better treatment alternatives. Patients with POTS might expect reduced symptoms and enhanced functional ability with the help of pharmacologic therapies. However, patient-specific treatment plans that account for comorbidities, drug tolerance, and other factors are necessary. Prescribing drugs to people with POTS might further complicate care if practitioners aren't aware of possible side effects and drug interactions. To get the best results from therapy, it's important to include non-pharmacologic measures alongside drug therapy [42].

Limitations

Studies included were either written in English or could be translated into English; therefore, non-English studies could have been neglected. The sample sizes used were relatively smaller with most studies with sample sizes between 20 and 80. This may have limited the statistical power of the studies, increased variability and the introduction of bias, and made it difficult to detect certain outcomes. Therefore, there is a need for more RCTs with larger sample sizes to improve the accuracy of the efficacy of medical interventions in managing postural tachycardia syndrome. The retrospective nature of this review integrating evidence from scholarly articles and not from actual patients limits the availability of some information, such as long-term adverse effects and follow-up of patients.

## Conclusions

There are significant challenges to studying POTS, and most published studies have a low sample size between 20 and 80. Pharmaceutical therapies have shown potential in managing POTS symptoms and enhancing patients' functional ability, but evidence remains weak due to studies being underpowered. Each patient's clinical presentation, comorbidities, and drug tolerance will dictate a unique treatment plan as this condition is heterogenous; hence, patient selection and enrolment have been major impediments to the development of therapies. More convincing evidence for the effectiveness of different pharmacologic therapies is required, and this can only be achieved via bigger, better-designed trials. There are significant challenges to studying POTS in a randomized controlled trial, and several trials have been terminated. Advances in diagnostic criteria, outcome measures, and patient selection are expected to improve the quality and relevance of future clinical trials.
